# Supplementary figures and images for: Outcomes of acute coronary syndrome patients with concurrent extra-cardiac vascular disease in the era of transradial coronary intervention: A retrospective multicenter cohort study
Source: PLoS One. 2019 Oct 16;14(10):e0223215. doi: 10.1371/journal.pone.0223215 (PMC6795465; doi:10.1371/journal.pone.0223215)

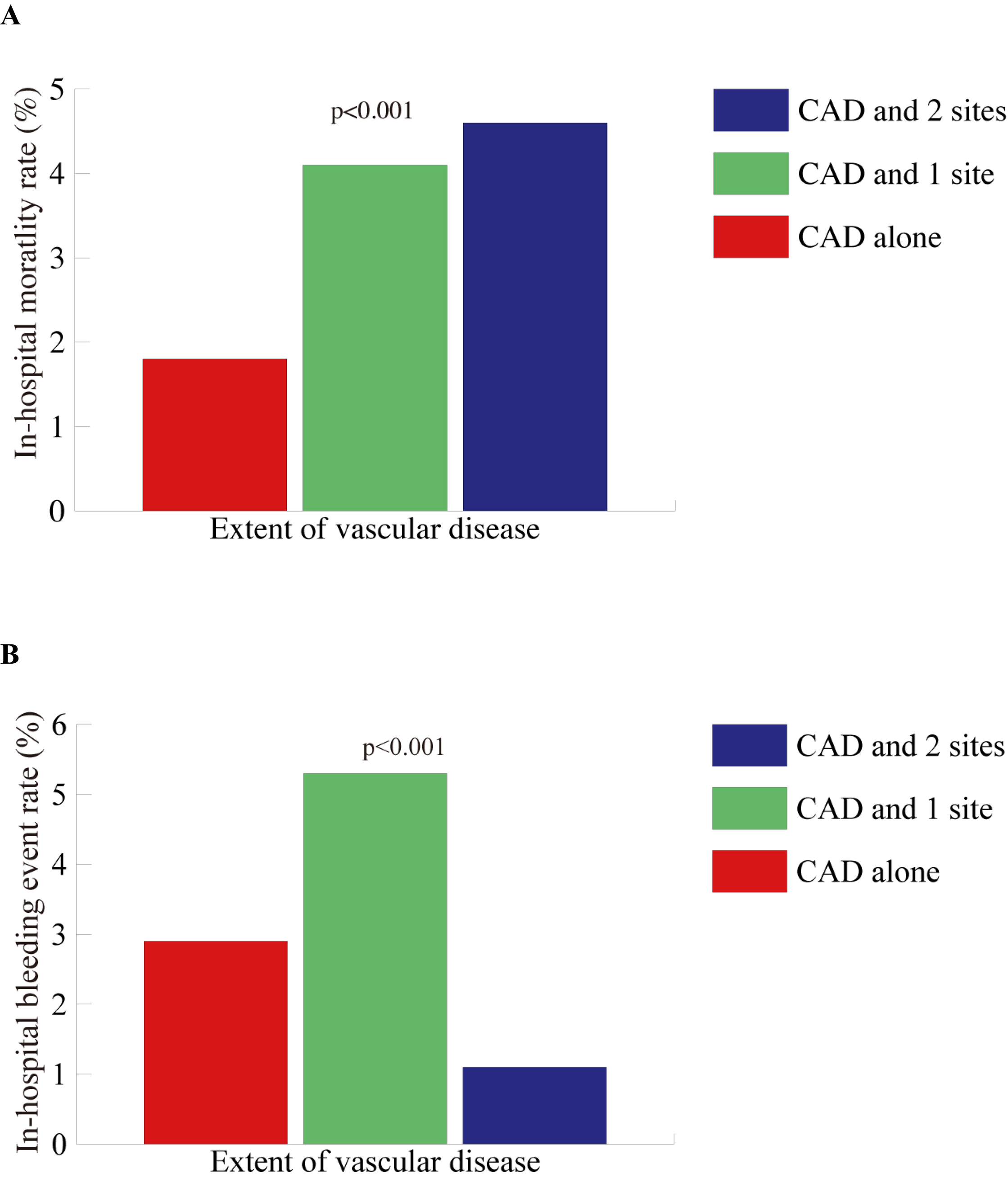

Supplement: S1 Fig — Bar chart demonstrating the rates of in-hospital deaths (A) and bleeding complications (B) in the three groups. ACS, acute coronary syndrome; CAD, coronary artery disease. (TIF) [file pone.0223215.s001.tif]

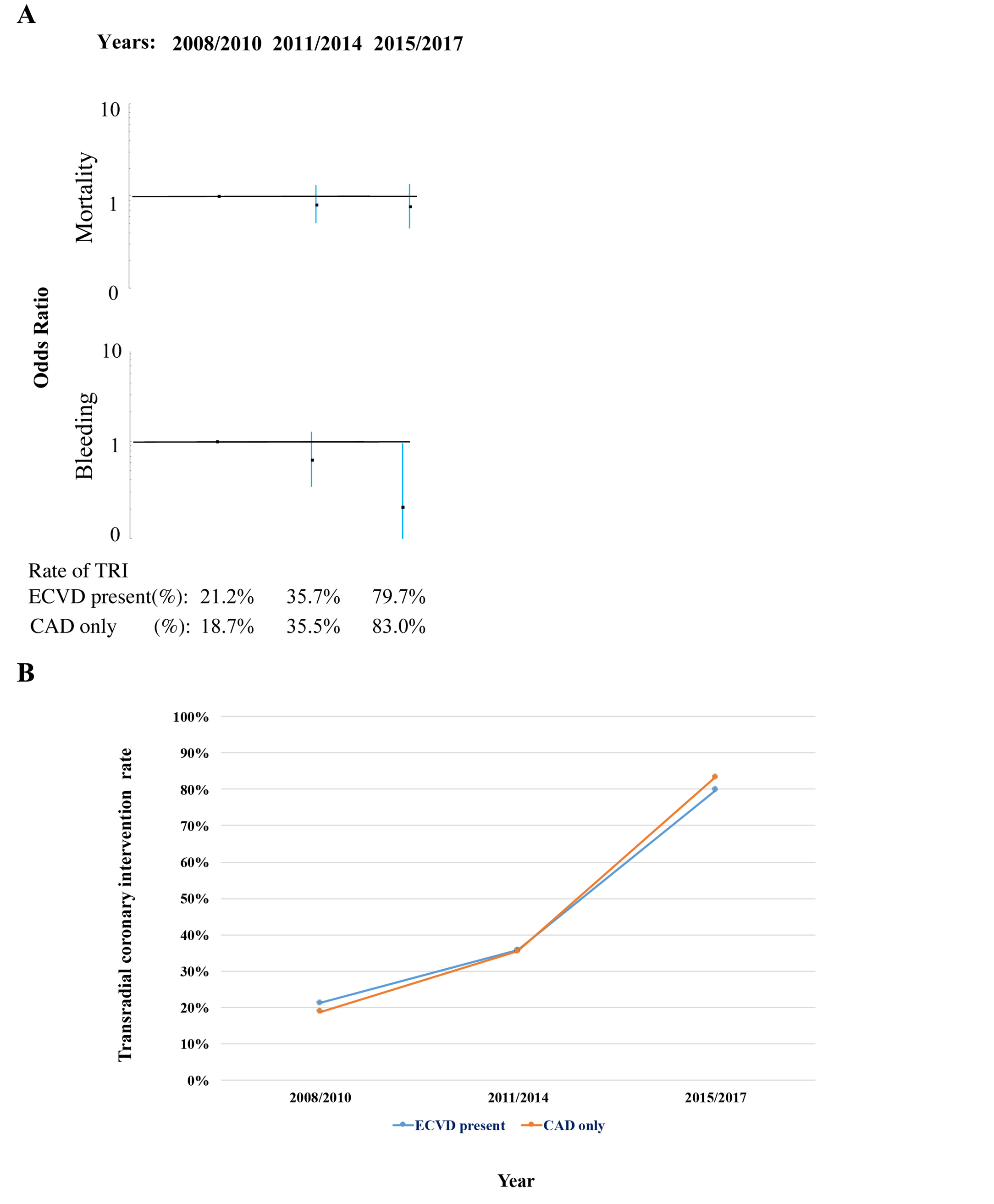

Supplement: S2 Fig — (A) Trends of in-hospital outcomes for patients with ACS and concomitant ECVDs in relation to the percentage of transradial intervention. Forest plots illustrate comparative outcomes with reference to the most recent years (2008–2010). (B) Trends in transradial intervention rate stratified by the presence of extra-cardiac vascular disease. Figure demonstrate the rate of transradial coronary intervention rate with or without extra-cardiac vascular disease, respectively. ACS, acute coronary syndrome; ECVD, extracardiac vascular disease; TRI, transradial intervention. (TIF) [file pone.0223215.s002.tif]

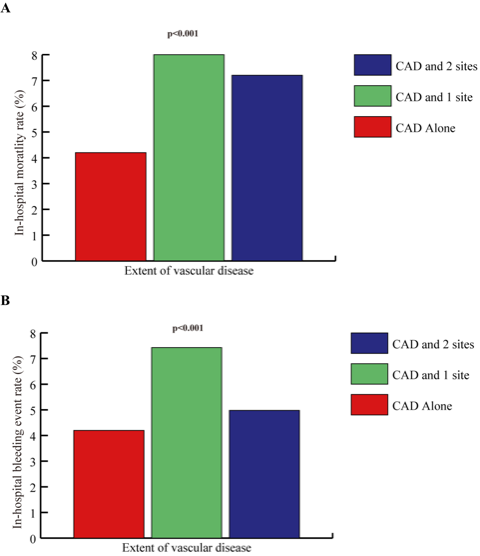

Supplement: S3 Fig — Bar chart demonstrating the rates of in-hospital deaths (A) and bleeding complications (B) in the three groups. ACS, acute coronary syndrome; CAD, coronary artery disease. (TIFF) [file pone.0223215.s003.tiff]

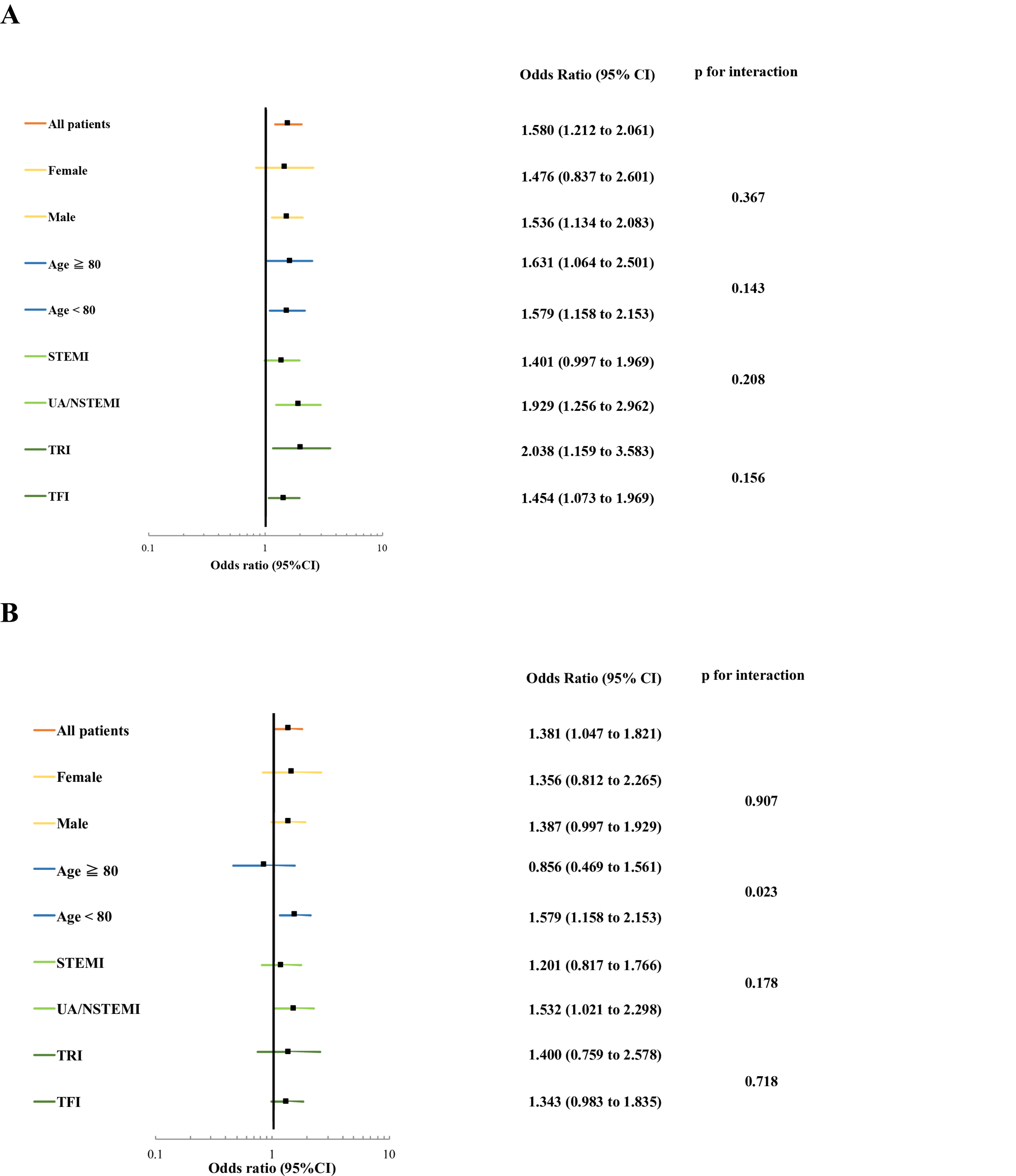

Supplement: S4 Fig — Comparison of various in-hospital mortality rates (A) and bleeding complication rates (B) among patients with and without extra-cardiac lesion. Forest plots demonstrate comparative outcomes of acute coronary syndrome patients between those with and without extra-cardiac lesions. CI, confidence interval; NSTEMI, non-ST-elevation myocardial infarction; STEMI, ST-elevation myocardial infarction; TFI, transfemoral intervention. TRI, transradial intervention; UA, unstable angina. (TIF) [file pone.0223215.s004.tif]

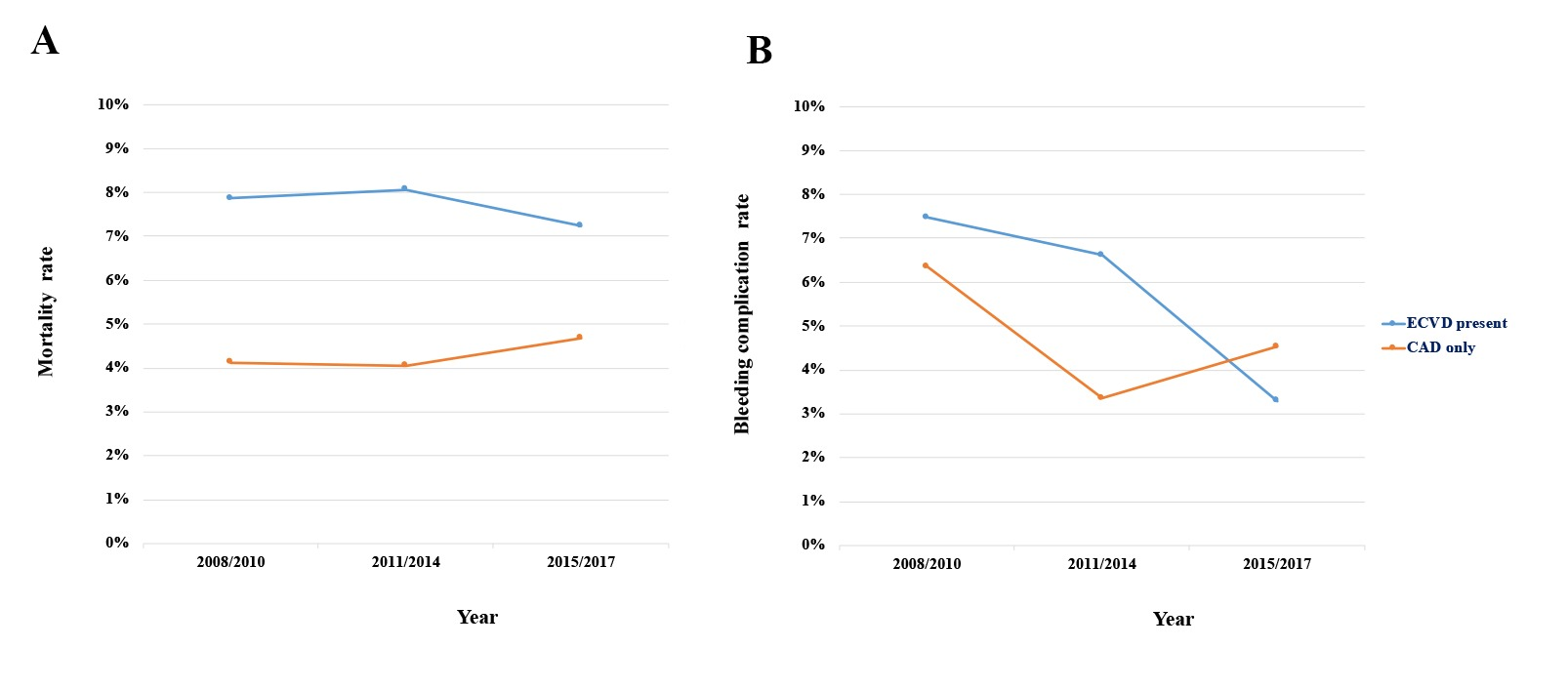

Supplement: S5 Fig — Figures demonstrate the in-hospital mortality (A) and bleeding complication (B) trends among patients with ACS with or without ECVD, respectively. ACS, acute coronary syndrome; CAD, coronary artery disease; ECVD, extracardiac vascular disease. (TIF) [file pone.0223215.s005.tif]
