# Supplementary material for: Outcomes of acute coronary syndrome patients with concurrent extra-cardiac vascular disease in the era of transradial coronary intervention: A retrospective multicenter cohort study
Source: PLoS One. 2019 Oct 16;14(10):e0223215. doi: 10.1371/journal.pone.0223215 (PMC6795465; doi:10.1371/journal.pone.0223215)
Supplement: S1 Table — BMI, body mass index; CAD, coronary artery disease; CABG, coronary artery bypass grafting; MI, myocardial infarction; NSTEMI, non-ST-elevation myocardial infarction; PCI, percutaneous coronary intervention; STEMI, ST-elevation myocardial infarction. (DOCX) [file pone.0223215.s006.docx]

**S1 Table.** Trends in clinical characteristics among patients with extra-cardiac lesion.

|  | 2008 / 2010 | 2011 / 2014 | 2015 / 2017 | p Value |
| --- | --- | --- | --- | --- |
| Female (%) | 51 (22.6%) | 127 (20.6%) | 31 (23.3%) | 0.864 |
| Age | 72.1 ± 9.9 | 73.1 ± 9.9 | 72.8 ± 11.1 | 0.463 |
| BMI | 23.3 ± 3.5 | 23.0 ± 3.6 | 22.8 ± 3.4 | 0.459 |
| History of MI (%) | 48 (21.2%) | 114  (18.5%) | 23 (17.3%) | 0.581 |
| History of heart failure (%) | 29 (12.8%) | 85 (13.8%) | 9  (6.8%) | 0.085 |
| Diabetes (%) | 103 (45.6%) | 272 (44.2%) | 56  (42.1%) | 0.620 |
| Dialysis (%) | 20 (8.8%) | 78 (12.7%) | 8  (6.0%) | 0.044 |
| Chronic kidney disease (%) | 127 (56.2%) | 361 (58.6%) | 75 (56.4%) | 0.662 |
| Chronic lung disease (%) | 10 (4.4%) | 40  (6.5%) | 8  (6.0%) | 0.531 |
| Hypertension (%) | 184 (81.4%) | 519 (84.3%) | 102 (76.7%) | 0.198 |
| Current smoker (%) | 60 (26.5%) | 215  (34.9%) | 32 (24.1%) | 0.01 |
| Dyslipidemia (%) | 131 (58.0%) | 400 (64.9%) | 70 (52.6%) | 0.013 |
| Family history of CAD (%) | 26  (11.5%) | 57  (9.3%) | 13  (9.8%) | 0.912 |
| Atrial fibrillation (%) | 3  (8.6%) | 212 (7.5%) | 116 (9.4%) | 0.662 |
| History of PCI (%) | 50 (22.1%) | 184  (29.9%) | 39 (29.3%) | 0.08 |
| History of CABG (%) | 23 (10.2%) | 23  (10.2%) | 4  (3.0%) | 0.038 |
| Intervention indication, STEMI (%) | 83 (36.7%) | 215  (34.9%) | 44 (33.1%) | 0.775 |
| Intervention indication, NSTEMI (%) | 44 (19.5%) | 123  (20.0%) | 34 (25.6%) | 0.312 |
| Heart failure at admission (%) | 42 (18.6%) | 132  (21.4%) | 21 (15.8%) | 0.28 |

BMI, body mass index; CAD, coronary artery disease; MI, myocardial infarction; PCI, percutaneous coronary intervention; CABG, coronary artery bypass grafting; STEMI, ST-elevation myocardial infarction; and NSTEMI, non-ST-elevation myocardial infarction.
