# Supplementary material for: Outcomes of acute coronary syndrome patients with concurrent extra-cardiac vascular disease in the era of transradial coronary intervention: A retrospective multicenter cohort study
Source: PLoS One. 2019 Oct 16;14(10):e0223215. doi: 10.1371/journal.pone.0223215 (PMC6795465; doi:10.1371/journal.pone.0223215)
Supplement: S2 Table — CTO, chronic total occlusion; DAPT, dual antiplatelet therapy; IABP, intra-aortic balloon pump; LAD, left arterial descending; LMT, left main trunk. (DOCX) [file pone.0223215.s007.docx]

**S2 Table.** Trends in procedural and prescription characteristics among patients with extra-cardiac lesion.

| Procedural characteristics | 2008 / 2010 | 2011 / 2014 | 2015 / 2017 | p Value |
| --- | --- | --- | --- | --- |
| Transradial (%) | 48 (21.2%) | 220  (35.7%) | 106 (79.7%) | <0.001 |
| Transfemoral (%) | 166 (73.5%) | 364  (59.1%) | 21 (15.8%) | <0.001 |
| IABP (%) | 21 (9.3%) | 57  (9.3%) | 8  (6.0%) | 0.471 |
| Bifurcation (%) | 46 (20.4%) | 147  (23.9%) | 38 (28.6%) | 0.207 |
| CTO (%) | 12 (5.3%) | 15  (2.4%) | 3  (2.3%) | 0.085 |
| Type C lesion (%) | 72 (31.9%) | 178  (28.9%) | 44 (33.1%) | 0.518 |
| Three vessel disease (%) | 88 (38.9%) | 190  (30.8%) | 36 (27.1%) | 0.033 |
| Target LMT (%) | 10 (4.4%) | 23  (3.8%) | 8  (6.0%) | 0.515 |
| Target LAD (%) | 95 (42.2%) | 253  (41.9%) | 54 (40.6%) | 0.953 |
| Cardio-protective medications |  |  |  |  |
| DAPT at  arrival (%) | 162 (71.7%) | 505  (82.0%) | 128 (96.2%) | <0.001 |
| DAPT at discharge (%) | 211 (93.4%) | 584  (94.8%) | 128 (96.2%) | 0.487 |
| Beta-blocker at discharge (%) | 156 (71.9%) | 426  (72.6%) | 85 (66.4%) | 0.372 |
| Statin at discharge (%) | 168 (77.4%) | 479  (81.5%) | 117 (91.4%) | 0.004 |

IABP, intra-aortic balloon pump; CTO, chronic total occlusion; LMT, left main trunk; LAD, left arterial descending; and DAPT, dual antiplatelet therapy.
