# Supplementary material for: Outcomes of acute coronary syndrome patients with concurrent extra-cardiac vascular disease in the era of transradial coronary intervention: A retrospective multicenter cohort study
Source: PLoS One. 2019 Oct 16;14(10):e0223215. doi: 10.1371/journal.pone.0223215 (PMC6795465; doi:10.1371/journal.pone.0223215)
Supplement: S3 Table — Modification of the effect of extra-cardiac vascular disease severity on in-hospital mortality (A) and bleeding complication (B) by procedural site when patients with cardiogenic shock and cardiopulmonary arrest were included. (A) Measure of interaction on additive scale: RERI (relative excess risk due to interaction) = -0.0528 (95% CI: -1.354 to 1.248); p = 0.9366. Measure of interaction on multiplicative scale: OR: 0.680 (95% CI: 0.399 to 1.158); p = 0.156. (B) Measures of interaction on additive scale: RERI = 0.383 (95% CI: -0.723 to 1.489); p = 0.497. Measure of interaction on multiplicative scale: OR: 2.005 (95% CI: 1.511 to 2.661); p<0.001. (DOCX) [file pone.0223215.s008.docx]

**S3A Table.** Modification of the effect of extra-cardiac vascular disease severity on in-hospital mortality by procedural site when patients with cardiogenic shock and cardiopulmonary arrest were included.

|  | Without extra cardiac lesion | | With extra cardiac lesion | |
| --- | --- | --- | --- | --- |
|  | Deceased/Alive, n | OR  (95% CI) | Deceased/Alive, n | OR  (95% CI) |
| Radial  Access | 52/2839 | 1.00 | 21/374 | 2.045  (1.192-3.511)  p=0.0094 |
| Femoral  Access | 260/4509 | 2.0154  (1.476-2.751)  p<0.0001 | 60/593 | 3.008  (2.035-4.447)  p<0.0001 |

Measure of interaction on additive scale: RERI (relative excess risk due to interaction) = -0.0528 (95% CI: -1.354 to 1.248); p=0.9366.

Measure of interaction on multiplicative scale: OR: 0.680 (95% CI: 0.399 to 1.158); p=0.156.

**S3B Table.** Modification of the effect of extra-cardiac vascular disease severity on in-hospital bleeding complication by procedural site.

|  | Without extra cardiac lesion | | With extra cardiac lesion | |
| --- | --- | --- | --- | --- |
|  | With/without bleeding complication, n | OR  (95% CI) | With/without bleeding complication, n | OR  (95% CI) |
| Radial  Access | 65/2826 | 1.00 | 14/381 | 1.375 (0.759-2.493)  p=0.294 |
| Femoral  Access | 257/4516 | 2.005  (1.512-2.661)  p<0.001 | 53/600 | 2.764  (1.893-4.034)  p<0.001 |

Measures of interaction on additive scale: RERI = 0.383 (95% CI: -0.723 to 1.489); p=0.497.

Measure of interaction on multiplicative scale: OR: 2.005 (95% CI: 1.511 to 2.661); p<0.001.
